# Targeted sampling reduces the uncertainty in force of infection estimates from serological surveillance

**DOI:** 10.3389/fvets.2022.754255

**Published:** 2022-07-28

**Authors:** Kiyeon Kim, Kimihito Ito

**Affiliations:** Division of Bioinformatics, International Institute for Zoonosis Control, Hokkaido University, Sapporo, Japan

**Keywords:** serological surveillance, wildlife, catalytic model, force of infection, confidence intervals, targeted sampling

## Abstract

Age bins are frequently used in serological studies of infectious diseases in wildlife to deal with uncertainty in the age of sampled animals. This study analyzed how age binning and targeted sampling in serological surveillance affect the width of the 95% confidence interval (CI) of the estimated force of infection (FOI) of infectious diseases. We indicate that the optimal target population with the narrowest 95% CI differs depending on the expected FOI using computer simulations and mathematical models. In addition, our findings show that we can substantially reduce the number of animals required to infer transmission risk by tailoring targeted, age-based sampling to specific epidemiological situations.

## Introduction

Serological surveillance monitors how fractions of individuals in a population have been infected with a specific infectious disease in the past or present. Serological tests analyze the level of pathogen-specific antibodies in the serum of individuals. Seroprevalence, the relative frequency of seropositive individuals in the population, helps us understand the epidemiology of the infectious disease, especially when the disease is asymptomatic or mildly symptomatic (1, 2). The data from serological surveillance are used to study the epidemiology of infectious diseases in humans (3), livestock (4), pet animals (5), and wildlife (6).

With mathematical models, seroprevalence data are analyzed to make an epidemiological inference. The basic reproduction number (*R*_0_) is one of the most vital epidemiological quantities, defined as the average number of secondary cases arising from a single infectious individual when it is exposed to a susceptible population (7). The force of infection (FOI) is another key epidemiological quantity, defined as the per capita rate at which susceptible individuals acquire infection (7). Various approaches have been applied to evaluate the FOI of endemic infectious diseases. Examples include the catalytic model assuming constant FOI within a homogeneously mixed population (8) and linear infection model with FOI changing linearly with age (9), the polynomial infection model that generalizes the linear infection model (10), and the exponentially damped linear model assuming an initially increasing FOI and an exponentially damping FOI with age (11). Using these models, the calculation of FOI requires the numbers of seropositive and seronegative individuals coupled with their age.

Currently, the age of domestic animals can be obtained in most countries. In contrast, the age of animals is inferred from age-dependent characteristics. For example, the age can be estimated by assessing animals' teeth (12–16). These approaches are applied after anesthetizing or killing the animals, and it is challenging to apply them to endangered animal species for their conservation. Some of their ages were obtained by tracking the animals from birth (17). Furthermore, measurement of size, weight, gum line recession, wear of a tooth, and tail length (18, 19) can be used to estimate the age of animals. Some studies have inferred animal age using the length of telomere (20) or the percentage of methylated DNA in specific genes (21).

Age binning is used in epidemiological studies of wildlife. It can be considered stratified sampling, which divides the population into homogeneous groups called stratum and collects samples from the strata (22). It is known that the variances of estimated quantities from stratified sampling are smaller than that from random sampling, especially when each stratum becomes homogeneous concerning the quantities under study. D' Amato et al. indicated that stratified sampling could reduce uncertainty in the mortality projections under a log bilinear Poisson Lee-Carter model (23). Age groups in wildlife studies are designed so that animals in the same age group share the same characteristics, such as physiology, behavior, and feeding, which affect disease transmission. The stratification of animals using their age may reduce the uncertainty in FOI since animals in the same bin are expected to have the same seroprevalence. Many studies use stratified sampling across animal ages for serological surveillance (24–27). Nevertheless, there is no preceding theoretical work studying the effect of stratified sampling on the confidence interval of FOI in serological surveillance.

This study analyzed how age binning and targeted sampling in serological surveillance affect the width of 95% CIs of the FOI of infectious diseases. We optimized the age group from which samples were drawn to minimize the width of 95% CI of the FOI. We assumed a situation where the exact ages of sampled animals are unknown and/or the age group from which sampling is allowed is limited. We show that using computer simulations and mathematical models, the optimal target population with the narrowest CI differs depending on the FOI. Moreover, we concluded by discussing the situations where surveillance targeting a specific age group is most beneficial.

## Materials and methods

### Model of seroprevalence

This study used a simple catalytic curve to model the seroprevalence of infectious diseases under endemic equilibrium (28). The catalytic model may not be common in the serological study of wildlife infection. Since our purpose is to investigate the effect of age binning on the CI of estimated FOI, we used this model to keep our mathematical model simple. The model assumes that (1) the population is homogeneously mixed, (2) FOI λ is constant over all age groups, and (3) infected individuals acquire a lifelong immunity (28). The proportion of seropositive individuals at age *a*, *p*(*a*), is approximated by a simple catalytic curve,


(1) $$\begin{array}{r} p\left(a\right)\approx 1-\exp\left(-\lambda a\right). \end{array}$$

Let *L* be the lifespan of the target animal under surveillance. An age group of animals can be represented by *G* (*qL, rL*), where *qL* and rL are the lower and upper bound ages of the age group, such that 0 ≤ *q* ≤ *r* ≤ 1. We set lifespan *L* to 150 weeks for the simulations and numerical analysis in this study. We assumed that all infected animals recover from their infections and none of them die of the infection. We also assumed that all animals die at an expected lifespan, called the Type 1 survival model. Therefore, the ages of animals in the population are uniformly distributed from 0 to *L*. Although these assumptions do not hold for wildlife populations, this simple model provided an analytically tractable means to evaluate the effect of targeted sampling on the CIs of estimated FOI.

### Simulation of serological surveillance

Serological surveillance focusing on an age group *G* (*qL, rL*) was simulated using samples made up of *N* animals whose ages were randomly chosen from a uniform real distribution ranging from *qL* to *rL*. Animal *i* at age *a*_*i*_ was set to be positive with probability *p* (*a*_*i*_) and negative with probability 1−*p* (*a*_*i*_) according to Eq. (1). We assume no error in diagnosis, that is, there is no false-positive or false-negative detection of pathogen-specific antibodies.

### Estimation of **λ*L***

From the ages of seropositive and seronegative animals, FOI λ can be estimated by maximizing the following likelihood function,


(2) $$\begin{array}{r} L\left(\lambda \;;a_{1},\ldots ,\;a_{k},\;a_{k+1},\ldots ,a_{N}\right)=\overset{k}{\underset{i=1}{\prod }}p\left(a_{i}\right)\overset{N}{\underset{i=k+1}{\prod }}\left(1-p\left(a_{i}\right)\right) \end{array}$$

where *a*_1_, …, *a*_*k*_ are ages of seropositive animals and *a*_*k*+1_, …*a*_*N*_ are ages of seronegative animals.

Let *p* (*qL, rL*) be the probability that an animal in *G* (*qL, rL*) is seropositive. From the uniform assumption on the age distribution, *p* (*qL, rL*) is given by


(3) $$\begin{array}{r} p\left(qL,rL\right)=\frac{\int_{qL}^{rL}p\left(t\right)dt\;}{\left(r-q\right)L}=1+\frac{\exp\left(-r\lambda L\right)-\exp\left(-q\lambda L\right)\;}{\left(r-q\right)\lambda L}. \end{array}$$

Let *k* be the number of seropositive samples among a total of *N* samples. The FOI λ can be estimated by maximizing the likelihood function of λ given by


(4) $$\begin{array}{r} L\left(\lambda \;;N,k\right)=\left(\begin{array}{c} N\\ k \end{array}\right)\;{\left(p\left(qL,rL\right)\right)}^{k}{\left(1-p\left(qL,rL\right)\right)}^{N-k}. \end{array}$$

In the case we draw samples from *m* age groups, the likelihood function is represented as a product of Eq. (4) as follows:


$$\begin{array}{r} L\left(\lambda \;;N_{1},\ldots ,N_{m},k_{1},\ldots ,k_{m}\right) \end{array}$$

(5) $$\begin{array}{rc} = & \overset{m}{\underset{i=1}{\prod }}\left(\begin{array}{c} N_{i}\\ k_{i} \end{array}\right)\;{\left(p_{pos}\left(q_{i}L,r_{i}L\right)\right)}^{k_{i}}{\left(1-p_{pos}\left(q_{i}L,r_{i}L\right)\right)}^{N_{i}-k_{i}} \end{array}$$

where, *q*_*i*_ and *r*_*i*_ are the ratios of the lower and upper bound ages of the *i*th age group to *L*.

When we assume that all samples are collected at the age of lifespan *L*, Eq. (1) can be used instead of Eq. (3), and we get


(6) $$\begin{array}{r} p\left(L,L\right)=1-\exp\left(-\lambda L\right). \end{array}$$

The likelihood function is represented by Eq. (4).

Note that the basic reproduction number, *R*_0_, can be calculated from the estimate of λ according to the formula given by Anderson and May (7) as follows:


(7) $$\begin{array}{r} R_{0}=\frac{\lambda L}{1-\exp\left(-\lambda L\right)}. \end{array}$$

If λ*L* is large enough, Eq. (7) can be reduced to *R*_0_ = λ*L*.

### Sampling strategies

To investigate how age binning and targeted sampling affect the width of 95% CIs of FOI in serological surveillance, we compared CIs of λ estimated under seven different sampling strategies (Table 1). We assume that the ages of animals in the target population do not affect the probability of animals being sampled for each strategy. Strategies 0, 1, and 2 collect samples randomly from the entire population, and *q* and *r* are set to zero and one. Strategy 0 represents the ideal surveillance, under which the exact age of each sampled animal is provided. Since the likelihood function in Eq. (2) needs the result of the serological test of animals coupled with their ages, Eq. (2) can be used to estimate λ from samples only for Strategy 0. In strategies 1 and 2, samples are drawn from the entire population, but the results of serological tests of sampled animals are summed up in the age group they belong. Strategy 1 divides the population into two groups at half of their lifespan. This strategy can be considered the standard approach using two age bins adopted for serological surveillance in wildlife. The likelihood function in Eq. (5) gives the likelihood function for Strategy 1 because it collects samples from two age groups. Strategy 2 treats the entire population as a single age group and does not provide age information for the animals sampled. Equation (4) with *q* = 0 and *r* = 1 gives the likelihood function for Strategy 2.

**Table 1:** Seven sampling strategy models.

**Strategy**	*q* ^*^	***r^†^***	**Age category**	**N**	**Sampling target and age information**
0	0	1	∞	100	Animals of all ages, exact age provided
1	0	1	2	100	Animals of all ages, two age bins, no exact age provided
2	0	1	1	100	Animals of all ages, no exact age provided
3	0	0.5	1	100	Young animals, no exact age provided
4	0.5	0.5	1	100	Animals whose age is half of the lifespan
5	0.5	1	1	100	Old animals, no exact age provided
6	1	1	1	100	Animals at dying

* *q, ratio of lower boundary age of the sampled age group to lifespan*.

† *r, ratio of upper boundary age of the sampled age group to lifespan*.

Strategies 3, 4, 5, and 6 collect samples only from animals with a specific age or a specific age group. Strategy 3 samples only animals with an age range from zero to 0.5*L*. Strategy 4 samples only animals at the age of 0.5*L*. Strategy 5 samples only animals whose age ranges from 0.5*L* to *L*. Strategy 6 samples only animals at the age of *L*, animals that are dying. Values of *q* and *r* for each strategy are shown in Table 1. Equation (5) with *q* = 0 and *r* = 0.5 gives the likelihood function for Strategy 3, and Eq. (5) with *q* = 0.5 and *r* = 1 gives that for Strategy 5. Equation (2) with *a*_*k*_= 0.5 for 1 ≤ *k* ≤ *N* gives the likelihood function for Strategy 4 and Eq. (2) with *a*_*k*_= 1 for 1 ≤ *k* ≤ *N* gives that for Strategy 6.

For each strategy, 1,000 simulations of serological surveillance were conducted for λ*L*= 1.5, 3.0, and 6.0. Values of λ*L* at 1.5, 3.0, and 6.0 are selected to represent a slightly transmissible infectious disease, for example, hepatitis E virus (29), moderately transmissible one, for example, African swine fever (30), and a highly transmissible one, for example, Bovine herpes virus 1 (31). The value of λ*L* and its 95% CI was estimated from the numbers of seropositive and seronegative samples drawn from target age groups. The estimates of λ*L* were calculated by maximizing the likelihood, and their CIs were calculated using profile likelihood method (32). More precisely, we calculated the intervals by finding λ*L* of which profile likelihood is log(*maximum likelihood*)−(χ^2^(1−0.05))/2 where the degrees of freedom of χ^2^ statistic is one. The width of CI was calculated by subtracting the lower bound of CI from the upper bound of CI. The width of 95% CI can be infinity and CI width of infinity were excluded from the calculation of average. We used three values for λ, 0.01, 0.02, and 0.04, of which λ*L* were 1.5, 3, and 6, respectively, to investigate the effect of λ on CI width of estimation.

### Effects of the target age group on estimation

We simulated serological tests using different values of *q* and *r* to examine the effect of *q* and *r* on the estimation of λ. We changed *q* from zero to one and *r* on *q* to one by a step of 0.02, where 0 ≤ *q* ≤ *r* ≤ 1. Estimates of λ*L* and their 95% CI width were estimated from samples and averaged over the 1,000 simulations. The upper and lower bounds of the 95% CI of λ*L* are calculated using the profile likelihood technique. The average width of the 95% CI was set to be undefined when more than 5% of 95% CI contained infinity.

### Age group that minimizes the expected width of 95% CI of **λ*L***

Parameter *q* or *r* was modified under the three constraints in Table 2 to evaluate the link between λ*L* and the target age group that minimizes the breadth of the 95% CI. The first constraint restricts the age of the target population to the animal at a specific age by *q* = *r* and changes *q*(= *r*). The second constraint fixes the lower bound *q* at zero and changes the upper bound *r*. The third constraint changes lower bound *q* under fixed upper bound *r*. Under constraints 1 and 3, we looked for the value of *q* having the narrowest width of 95% CI of λ*L* for 0 ≤ λ*L* ≤ 10. Under constraint 2, we looked for the value of *r* having the narrowest width of 95% CI. *N* is the number of animals sampled, and it is set to 50, 100, or 200.

**Table 2:** Three constraints for calculating the expected width of 95% CI of λ*L*.

**Constraint**	**Minimum sampling age**	**Maximum sampling age**
Constraint 1	*qL*	*qL*
Constraint 2	0	*rL*
Constraint 3	*qL*	*L*

### Analytical approach to the expected width of 95% CI

Under the three constraints in Table 2, we can calculate the expected values of the 95% CI width of estimated λ*L* using the normal approximation of the binomial distribution. Suppose the number of seropositive animal observations follows a binomial distribution with sample size *N* and seroprevalence *p*. Assuming that *N* is the number of samples and large enough, the number of seropositive animals, *k*, can be approximated by a normal distribution with a mean of *Np* and variance of *Np* (1−*p*). In total, 95% of *k* will be within the following range:


(8) $$\begin{array}{r} Np-1.96\sqrt{Np\left(1-p\right)}\leq k\leq Np+1.96\sqrt{Np\left(1-p\right)}. \end{array}$$

Let $\hat{p}=k/N\;$be empirical seroprevalence estimated from samples, and then we obtain the following relationship:


(9) $$\begin{array}{r} p-1.96\sqrt{p\left(1-p\right)/N}\leq \hat{p}\leq p+1.96\sqrt{p\left(1-p\right)/N}. \end{array}$$

If Inequality (9) is rearranged for *p*, we obtain the following relationship:


(10) $$\begin{array}{r} \hat{p}-1.96\sqrt{\frac{\hat{p}\left(1-\hat{p}\right)}{N}}\leq p\leq \hat{p}+1.96\sqrt{\frac{\hat{p}\left(1-\hat{p}\right)}{N}}. \end{array}$$

The seroprevalence, *p*, at a specific age (an age group under Constraint 1) is calculated from Eq. (1). The estimated value of λ*L* can be calculated as follows:


(11) $$\begin{array}{r} \lambda L=-\frac{\ln\left(1-p\right)}{q}. \end{array}$$

From Inequality (10) and Eq. (11), we get the following relationship:


$$\begin{array}{rc} - & \frac{1}{q}\ln\left(1-\hat{p}+1.96\sqrt{\frac{\hat{p}\left(1-\hat{p}\right)}{N}}\right)\leq \lambda L \end{array}$$

(12) $$\begin{array}{r} \leq -\frac{1}{q}\ln\left(1-\hat{p}-1.96\sqrt{\frac{\hat{p}\left(1-\hat{p}\right)}{N}}\right). \end{array}$$

The width of 95% CI of λ*L* can be estimated by subtracting the lower boundary of 95% CI of λ*L* from the upper boundary of 95% CI of λ*L* below:


(13) $$\begin{array}{r} \frac{1}{q}\left(\ln\left(\frac{1-\hat{p}+1.96\sqrt{\hat{p}\left(1-\hat{p}\right)/N}}{1-\hat{p}-1.96\sqrt{\hat{p}\left(1-\hat{p}\right)/N}}\right)\right). \end{array}$$

Since the expected value of $\hat{p}$ is *p*, then the expected value of 95% CI width of λ*L* is as follows:


(14) $$\begin{array}{r} \frac{1}{q}\left(\ln\left(\frac{1-p+1.96\sqrt{p\left(1-p\right)/N}}{1-p-1.96\sqrt{p\left(1-p\right)/N}}\right)\right). \end{array}$$

Substituting *p* with 1−exp(−λ*Lq*) the expected width of 95% CI is represented as


(15) $$\begin{array}{r} \frac{1}{q}\left(\ln\left(\frac{\exp\left(-\lambda Lq\right)+1.96\sqrt{\left(1-\exp\left(-\lambda Lq\right)\right)\exp\left(-\lambda Lq\right)/N}}{\exp\left(-\lambda Lq\right)-1.96\sqrt{\left(1-\exp\left(-\lambda Lq\right)\right)\exp\left(-\lambda Lq\right)/N}}\right)\right). \end{array}$$

The seroprevalence, *p*, at a specific age group under constraints 2 and 3 is calculated from Eq. (3). However, the analytical derivation of an explicit form of λ*L* from Eq. (3) is complicated, and we get λ*L* using numerical analysis of Eq. (3). The expected width of 95% CI of λ*L* was estimated using Eq. (10) in the same way as Constraint 1.

## Results

### Estimation by different strategies

Table 3 displays estimates of over 1,000 repeats of serological surveillance simulations, estimations of λ*L* with 95% CIs, and the width of 95% CIs. When λ*L* is 1.5, all strategies estimated λ*L* close to the true value of 1.5. However, the average width of their CI showed variations among strategies. Strategies 0, 1, and 2, all sampled from the entire population, had different CI widths. This difference is attributed to the difference in information given to each strategy. Strategy 0, given the complete age information of samples, had the narrowest CI width among the three. Strategy 2, given no age information of samples, had the widest CI width. Strategy 1 was given incomplete age information, whether each animal was younger (or older) than half of their lifespan, and ranked the second of the three.

**Table 3:** Estimated values of λ*L* and their 95% CIs for seven sampling strategies.

**λ*L***	**Strategy**	***q*** ^*^	*r* ^^†^^	**Age category**	**Estimated λ*L* (95% CI)**	**CI width**
1.5	0	0	1	∞	1.512 (1.112, 2.007)	0.895
	1	0	1	2	1.510 (1.102, 2.026)	0.924
	2	0	1	1	1.514 (1.078, 2.105)	1.027
	3	0	0.5	1	1.512 (1.009, 2.191)	1.182
	4	0.5	0.5	1	1.512 (1.136, 1.973)	0.837
	5	0.5	1	1	1.512 (1.159, 1.942)	0.784
	6	1	1	1	1.525 (1.186, 1.936)	0.751
3.0	0	0	1	∞	3.049 (2.300, 3.994)	1.694
	1	0	1	2	3.082 (2.288, 4.136)	1.848
	2	0	1	1	3.054 (2.174, 4.378)	2.204
	3	0	0.5	1	3.026 (2.154, 4.206)	2.052
	4	0.5	0.5	1	3.048 (2.370, 3.871)	1.500
	5	0.5	1	1	3.050 (2.334, 3.979)	1.645
	6	1	1	1	3.096 (2.310, 4.229)	1.920
6.0	0	0	1	∞	6.194 (4.477, 8.610)	4.133
	1	0	1	2	6.270 (4.328, 9.717)	5.389
	2	0	1	1	6.334 (4.116, 10.724)	6.609
	3	0	0.5	1	6.173 (4.390, 8.862)	4.472
	4	0.5	0.5	1	6.149 (4.592, 8.388)	3.796
	5	0.5	1	1	5.850 (4.030, 9.231)^§^	5.200
	6	1	1	1	4.548 (3.112, 7.319)^�^	4.207

*Simulations were repeated 1,000 times for each sampling strategy, and the estimated values, their 95% CIs, and the width of 95% CIs were averaged over repeated simulations*.

* *q: ratio of the minimum boundary age of the sampled age group to lifespan*.

† *r: ratio of the maximum boundary age of the sampled age group to lifespan*.

§ *The estimation of 95% CI was averaged only for 767 successful simulations out of 1,000*.

� *The estimation of 95% CI was averaged only for 201 successful simulations out of 1,000*.

Among all strategies, Strategy 6, which takes samples only from animals at the age of death, had the narrowest 95% CI of 0.751. The second narrowest value was 0.784 with Strategy 5, which takes samples only from animals whose ages are older than half of their lifespan. These results are counter-intuitive because CI estimated from a subpopulation was narrower than Strategy 0; random sampling from the entire population with complete age information resulted in a width of 0.895. The maximum average width of CI of λ*L* was 1.182 with Strategy 3, which takes samples only from animals whose ages are younger than half of their lifespan. When λ*L* = 3.0, the seven tested strategies estimated λ*L* close to the true value of 3.0. Again, the average widths of their CI were different depending on strategies (Table 3), but the tendency was different from when λ*L* = 1.5. Among strategies, Strategy 4, which takes samples only from the animal at the age of half of the lifespan, had the narrowest 95% CI of 1.500. The second narrowest is Strategy 5, which takes samples only from old animals older than half of their lifespan. Again, these findings are counter-intuitive because the CIs are narrower than those of Strategy 0. In sampling Strategy 2, the largest average width of CI of λ*L* was 2.204.

When λ*L* = 6.0, all strategies except Strategy 6 estimated λ*L* close to the true value of 6.0 (Table 3). Among all strategies, Strategy 4, which takes samples only from the animal at half of the lifespan, again had the narrowest 95% CI of 3.796. The second narrowest is Strategy 0, which takes samples from animals at any age with complete age information. Seroprevalence in the old population is close to one, and the information from animals in the old population becomes uninformative. For this reason, the FOI estimated by Strategy 6 became inaccurate. Moreover, the width of CI of λ*L* of the Strategies 5 and 6 was not available for some simulations because λ*L* is estimated to be infinity when only seropositive animals are sampled.

### Effect of target age group on estimation

**Figure 1:**
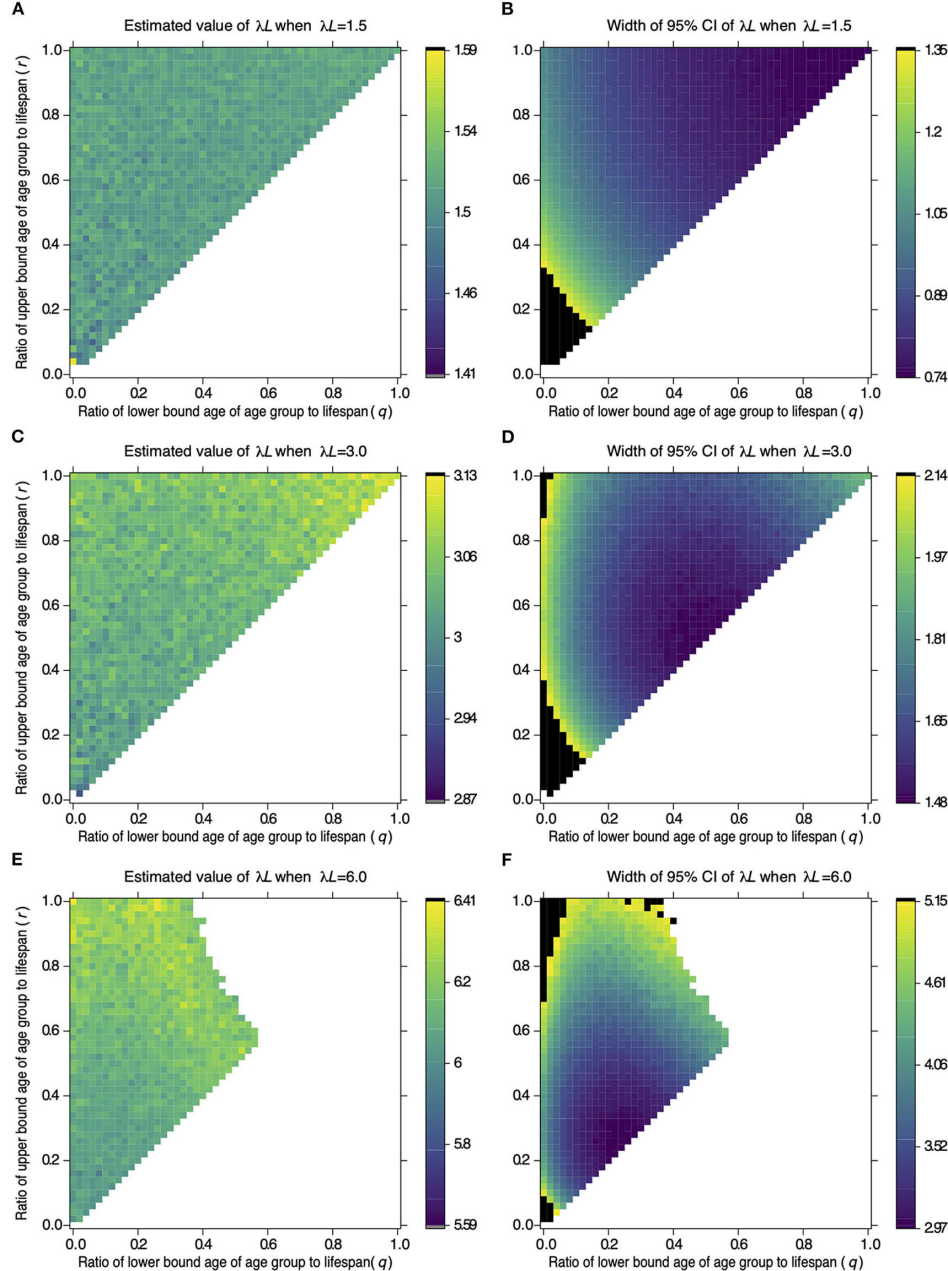
Effect of the target age group on the estimate of λ*L* and its 95% CI width. The color of a cell in **(A,C,E)** represents the value of λ*L* estimated from samples of the target age group defined by age parameters *q* and *r* on the *x*- and *y*-axis. A cell in **(B,D,F)** represents the width of 95% CI of λ*L* estimated from the samples as **(A,C,E)**, respectively. Values of λ*L* and 95% CI width were estimated from 100 samples drawn from the target population. Serological surveillance was simulated 1,000 times under settings where λ*L* = 1.5 **(A,B)**, λ*L* = 3.0 **(C,D)**, and λ*L* = 6.0 **(E,F)**. The color of a cell represents the averaged value in 1,000 repetitions, and the color key next to each panel shows colors associated with the values. The black cells in **(B,D,F)** represent widths above the 95th percentile of all widths of CI. The white cells represent the combination of sampling age parameters. The results are not available to estimate 95% CI of λ*L* by the profile likelihood method in more than 5% of repetitions.

Figure 1 shows the estimates and widths of 95% CI of λ*L* estimated from samples drawn from different age groups in serological surveillance simulations. The top left corner, where *q* = 0 and *r* = 1 in each panel, represents Strategy 2 in Table 1. The point at the middle of the left edge, where *q* = 0 and *r* = 0.5, represents Strategy 3, and the point at the center of the top edge, where *q* = 0.5 and *r* = 1, represents Strategy 5. The top right corner, where *q* = 1 and *r* = 1, represents Strategy 6, and the point in the center of the panel, where *q* = 0.5 and *r* = 0.5, represents Strategy 4.

Figures 1A,B indicates that the estimates have the narrowest 95% CI in the area around the top right corner when λ*L* is 1.5. This indicates that sampling from an old population is reliable when λ*L* is 1.5. The reliable area changes when λ*L* is 3.0 or 6.0. Estimates have the narrowest 95% CI in the area around the center of the diagonal line when λ*L* is 3.0 (Figures 1C,D). The reliable area shifts to the lower left when λ*L* is 6.0 (Figures 1E,F). The width of 95% CI became large for some combinations of *q* and *r* (black cells in Figures 1B,D,F. The widths of 95% CI were not available for other combinations (white cells in Figures 1B,D,F). These points are addressed in the discussion.

Table 4 shows the combination of *q* and *r*, which resulted in the minimum width of 95% CI of λ*L* when λ*L* is 1.5 or 3.0 or 6.0 in serological surveillance simulations. These results suggested that the narrowest 95% CI can be achieved by surveillance targeting the old population when λ*L* is small and surveillance targeting the young population when λ*L* is large. Estimations and 95% CI for combinations of *q* and *r* when λ*L* were 1.5, 3.0, and 6.0 are provided in Supplementary Tables 1–3, respectively.

**Table 4:** Combinations of *q* and *r* resulted in the narrowest 95% CI of λ*L*.

λ*L*	***q***	***r***	**Width of 95% CI of** λ*L*
1.5	0.98	0.98	0.741
3.0	0.48	0.48	1.485
6.0	0.22	0.26	2.974

**Figure 2:**
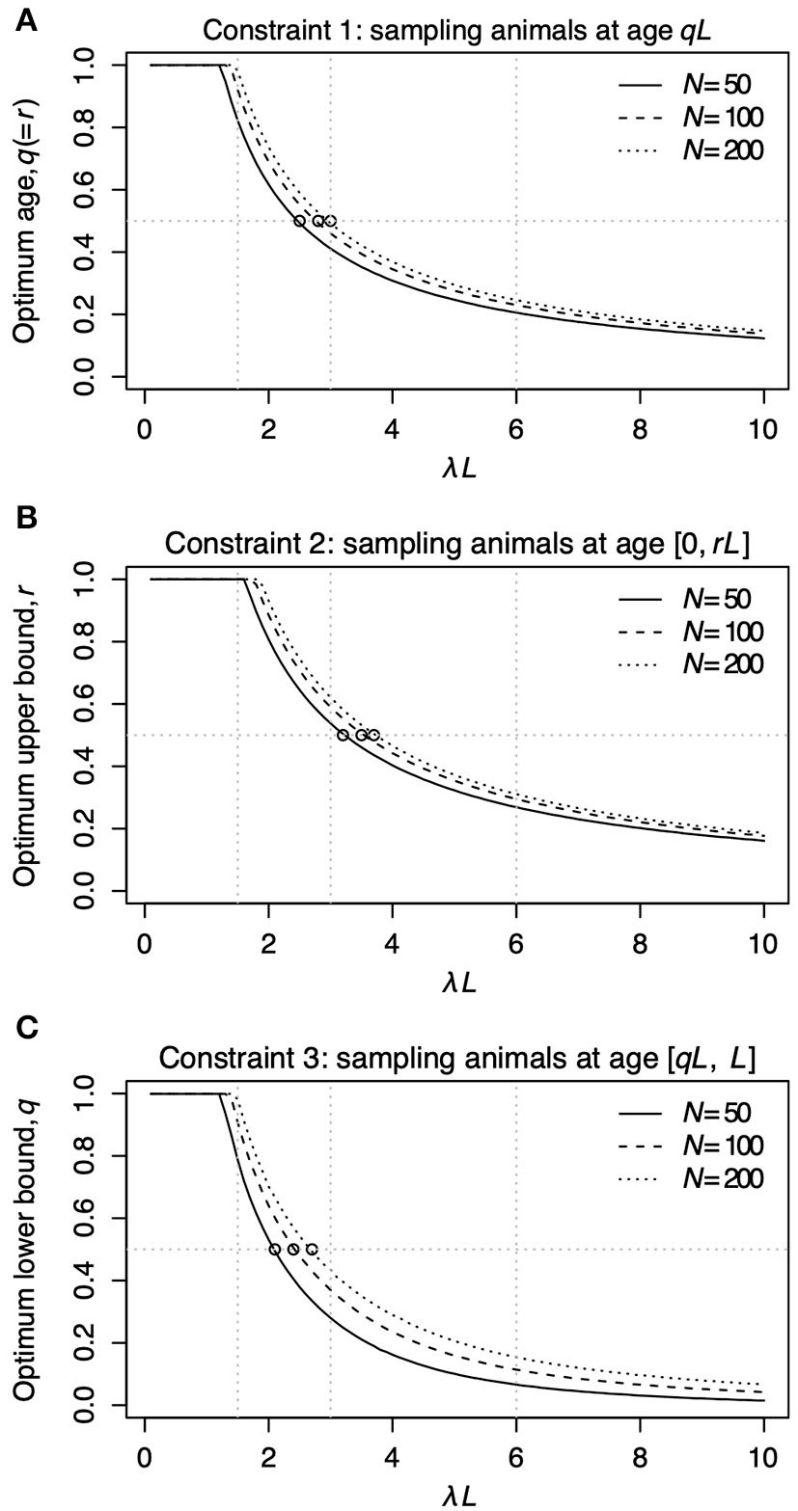
The relationship between λ*L* and the optimum age parameter value for Constraints 1 **(A)**, 2 **(B)**, and 3 **(C)**. In each panel, the solid line represents *N* = 50, the dashed line represents *N* = 100, and the dotted line represents *N* = 200. The horizontal line in **(A)** represents *r* = *q* = 0.5 for Constraint 1. The horizontal line in **(A)** represents *r* = 0.5 for Constraint 2. The horizontal line in **(A)** represents *q* = 0.5 for Constraint 3. The open circles in **(A–C)** denote the points where the optimum sampling age parameters are 0.5. Vertical panel lines **(A–C)** are drawn at λ*L* = 1.5, λ*L* = 3.0, and λ*L* = 6.0, which are the values used in the sampling simulation in Table 3.

Figure 2 shows the relationship between λ*L* and the sampling age parameter that resulted in the narrowest 95% CI. We call such a parameter value an optimum age parameter value. The optimum age parameter values for all three Constraints were one when λ*L* is small (Figures 2A–C). The range of λ*L* at the height of one indicates that the inclusion of the oldest members of the population is needed to minimize the width of 95% CI of λ*L* when λ*L* is small. For all Constraints in Table 2, the optimum age parameter values decreased as λ*L* increased (Figures 2A–C). Table 5 shows the values of λ*L* and their widths of 95% CI when the optimum age parameter value is 0.5 for each Constraint. The optimum age parameter value for each Constraint is older than half of the lifespan if λ*L* is less than the value indicated in Table 5 and *vice versa*.

**Table 5:** The value of λ*L* and the width of 95% CI of estimated λ*L* when the optimum age parameter values are 0.5.

**Constraints**	***N***	λ*L*	**Width of 95% CI**
Constraint 1 (*q* = 0.5, *r* = 0.5)	50	2.46	1.846
	100	2.76	1.408
	200	2.95	1.041
Constraint 2 (*q* = 0, *r* = 0.5)	50	3.22	3.183
	100	3.54	2.397
	200	3.73	1.756
Constraint 3 (*q* = 0.5, *r* = 1)	50	2.08	1.649
	100	2.43	1.320
	200	2.67	1.008

**Figure 3:**
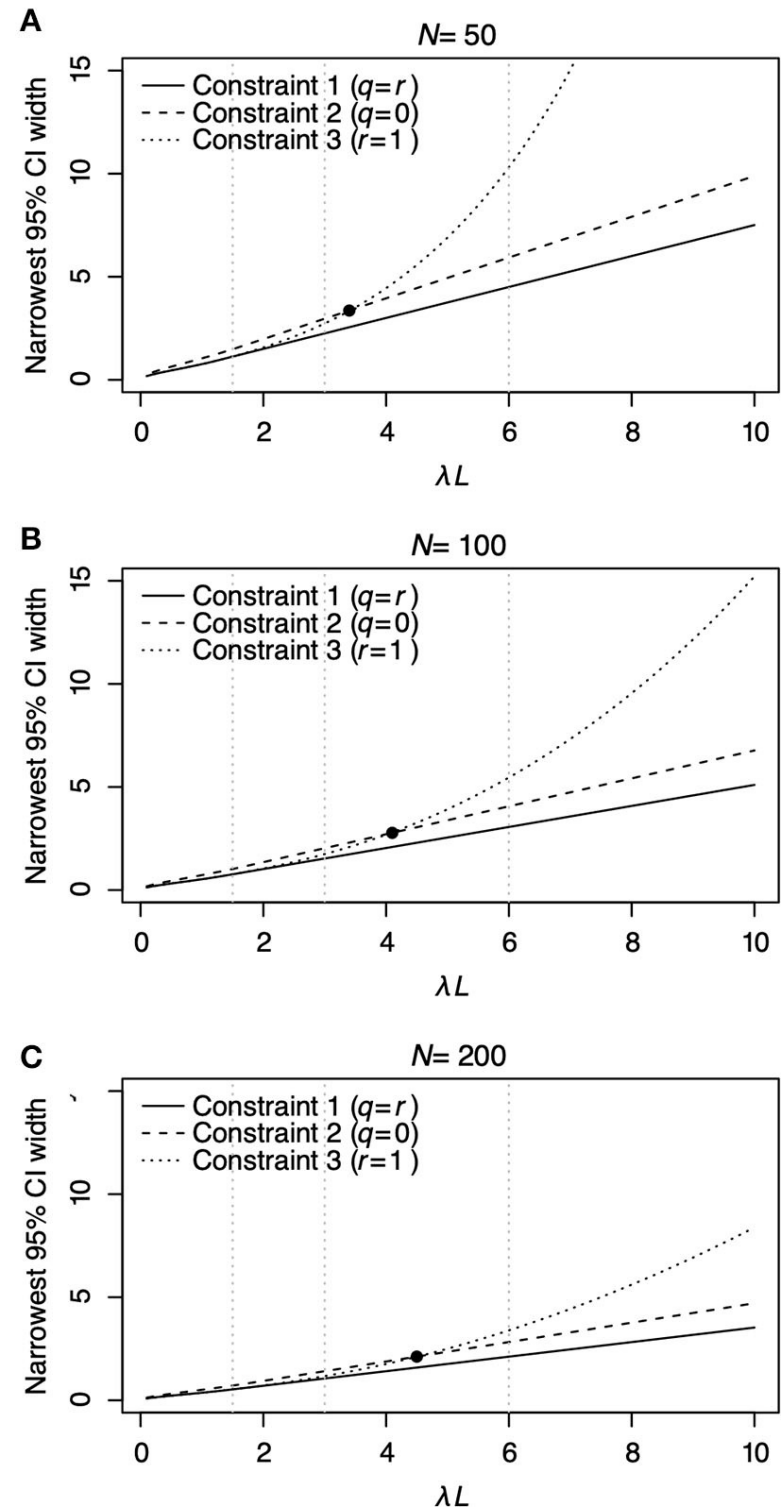
The relationship between λ*L* and the narrowest 95% CI of λ*L* for each constraint is shown in Table 2 under different *N* = 50 **(A)**, *N* = 100 **(B)**, and *N* = 200 **(C)**. In each panel, the solid line represents Constraint 1, where *q* = *r*; the dashed line represents Constraint 2, where *q* = 0; and the dotted line represents Constraint 3, where *r* = 1. The filled circles in **(A–C)** denote the point where the narrowest 95% CI of λ*L* under Constraints 2 and 3 has the same value. Vertical panel lines **(A–C)** are drawn at λ*L* = 1.5, λ*L* = 3.0, and λ*L* = 6.0, which are the values used in the surveillance simulations in Table 3.

Figure 3 shows the relationship between λ*L* and the narrowest width of 95% CI of estimated λ*L*. The narrowest 95% CI of λ*L* monotonically increases as λ*L* increases for all Constraints regardless of the sample number *N* (Figure 3). Among the three constraints, Constraint 1 (solid line) has the narrowest width of 95% CI for all λ*L* regardless of *N*. Comparing Constraints 2 (dashed line) and 3 (dotted line), Constraint 2 has a narrower width of 95% CI than Constraint 3 when λ*L* ≤ 3.43, λ*L* ≤ 4.05, and λ*L* ≤ 4.52 for *N* = 50, 100, and 200, respectively. Constraint 3 has a narrower width of 95% CI than Constraint 2 when λ*L* > 3.43, λ*L* > 4.05, and λ*L* > 4.52 for *N* = 50, 100, and 200, respectively. The slope of the curve of Constraint 3 increases by a large amount compared with that of constraints 1 and 2. This result indicates that Constraint 3, where *r* = 1, should be carefully selected to estimate λ*L* when λ*L* > 3.43, λ*L* > 4.05, and λ*L* > 4.52 for *N* = 50, 100, and 200, respectively.

Supplementary Figure 1 shows the relationship between the age parameter *q* [panels (A–C) and (G), (H), and (I) ]or *r* [panels (D), (E), and (F)] and the expected width of 95% CI of λ*L*. The narrowest width of 95% CI of λ*L* decreases as λ*L* decreases. Supplementary Figure 1 shows how the sample number affects the width of 95% CI of λ*L*. The expected width of 95% CI of estimated λ*L* decreased as the number of samples increased.

## Discussion

This study analyzed how age binning and targeted sampling affected the accuracy of the estimation of the FOI, λ. Assuming that infectious diseases are endemic in a homogeneously mixed population with constant λ over age under lifelong immunity, we found that the age group that minimizes the width of 95% CI of λ*L* was different depending on the value of λ and the number of samples *N*.

Using simulations of serological surveillance, we found that surveillance targeting a specific age or age group resulted in a narrower 95% CI of estimated λ*L* than the standard age-binned sampling from the entire population (Strategy 1) in particular situations (Table 3). Surveillance targeting animals older than half of their lifespan (Strategy 5) had a narrower 95% CIs than not only a standard wildlife surveillance approach targeted the entire population using two age bins (Strategy 1) but also well-informed surveillance that targeted the entire population with precise age (Strategy 0) and when λ*L* = 1.5 (Table 3). Surveillance targeted animals younger than half of their lifespan (Strategy 3) had a narrower 95% CIs than Strategy 1 when λ*L* = 6.0 (Table 3). These results indicated a chance to narrow down the 95% CI by serological surveillance targeting a particular age group.

Even when animals' actual ages were provided (Strategy 0), surveillance targeting the entire population did not always guarantee the calculation of the narrowest width of the 95% CI of λ*L* (Table 3). These results are related to the properties of stratified sampling. Targeted serological surveillance sampling can be considered a special case of stratified sampling. Stratified sampling can reduce uncertainty compared with a random sampling of the entire population (22). Seroprevalence in the young population is mostly zero, and seroprevalence in the old population is more informative than that in the young population when FOI is small. When FOI is large, on the other hand, the seroprevalence in the old population is close to one, and the seroprevalence in the young population is more informative than that in the old population. Therefore, more samples from the old population can be used by Strategies 5 and 6 than by Strategy 0 or 1. This is why, when FOI is modest, tailored sampling outperforms fully informed sampling from the entire population.

Constraint 1, surveillance targeting animals at a given age, was found to have the smallest 95% CI of λ*L* regardless of λ*L* and three tested *N* among the three constraints in Table 2. Nevertheless, it is difficult to conduct surveillance targeting only animals of a specific age in wildlife. When Constraint 2, surveillance targeting animals younger than specific age, and Constraint 3, surveillance targeting animals older than specific age, are compared, the choice between Constraints 2 and 3 depends on the value of λ*L*. For example, when the sample number *N* = 100, surveillance based on Constraints 2 and 3 intersects at λ*L* = 4.05. Constraint 3 has a narrower 95% CI than Constraint 2 when λ*L* is less than the intersection and *vice versa*. Moreover, note that the width of the narrowest 95% CI of Constraint 3 is much wider than that of Constraint 2 in the right area of Figure 3B. These findings showed that surveillance targeting animals older than a specified age (Constraint 3) is a good choice when the value of λ*L* is less than the intersection point. Still, it is not a good decision when the value of λ*L* is greater than the intersection point.

Extremely broad confidence intervals, including ones with infinite breadth, are produced by some combinations of λ*L* and target age groups. The estimate of λ*L* becomes infinity when all samples are seropositive in a surveillance simulation. As a result, the 95% CI of estimated λ*L* can have an infinite width. This phenomenon was observed as white cells in the upper right corner in Figure 1F, where old animals were sampled when λ*L* = 6.0. Alternatively, most samples can be seronegative when λ*L* is small and surveillance targets the young age group. As a result, the 95% CI of estimated λ*L* becomes wide. The confidence interval can also be wide; this phenomenon was observed as black cells at the lower left corner in Figures 1B,D, where young animals were sampled when λ*L* = 1.5 or λ*L* = 3.0. These phenomena can also be observed in Supplementary Figure 1.

Generally, the width of 95% CI of λ*L* can be narrowed down by increasing the number of samples. However, collecting serum samples from wildlife is limited, particularly for endangered species. Our results indicated that the width of the 95% CI of FOI can be narrowed down by increasing the number of samples in specific age ranges. Setting the age group to be sampled at the design step of surveillance can reduce the number of sampled animals in particular situations. For example, the standard sampling strategy using two age bins from the entire population (Strategy 1) with 100 serum samples had a 95% CI width of λ*L* of 0.924 on average under λ*L* = 1.5 (Table 3). Surveillance only from animals older than half their lifespan (Strategy 5) with 73 samples resulted in a 95% CI of 0.924 (Supplementary Figure 2). Switching the sampling strategy from strategies 1 to 5 can decrease the number of sampled animals in this situation.

In this study, we assumed that the age distribution in the population was uniform, which is rarely true for wild animals. A non-uniform age distribution can affect Eq. (3) and its subsequent derivations. However, we think the uniform age assumption does not affect our main results, as long as the seroprevalence remains similar. In addition, we assumed that all animals recovered from the infectious disease without dying of infection. The model can be used to analyze infectious diseases in which the lethality is limited if the seroprevalence is not affected by fatal infections. This non-fatal assumption may be critical if a fatal infectious disease is analyzed because the seroprevalence of old animals does not increase in the same way as a non-fatal infectious disease. This is a common difficulty in analyzing seroprevalence data of a fatal infectious disease, and our presented method does not apply to serological surveillance of fatal infectious diseases.

We used the simplest model of seroprevalence called the catalytic model, which assumes that FOI is constant over age in a homogeneously mixed population acquiring lifelong immunity (8). However, several models assume that the FOI can depend on age. Among them are the catalytic linear infection model (11), the catalytic polynomial infection model (10), and the exponentially damped linear model (9). Furthermore, FOI could be represented using the Who Acquires Infection From Whom matrix, which represents transmissibility among age groups (7, 33, 34). Optimizing targeted sampling in serological surveillance under these models remains our future work.

The results in this study are based on computer simulations of serological surveillance. Justifying our method using real datasets of serological surveillance is another direction of our future work. When applying our technique to real datasets, the number of samples, sampling times, and types of pathogens, such as bacteria and viruses, are all significant considerations.

In the serological surveillance of endemic disease, we discovered that tailored sampling could lower the width of the 95% CI of FOI. To take full advantage of the targeted sampling, however, it is necessary to know the expected value of FOI of the target infectious disease in advance. Estimating FOI itself is the purpose of serological surveillance, and it is difficult to know the expected value of FOI. One realistic solution to this problem is to apply targeted sampling after estimating FOI using preliminary serological surveillance with few samples. FOI estimated from previous research can be used for deciding the target range of samples for current surveillance. This approach can be considered adaptive surveillance (35), where surveillance is designed based on the results of previous modeling studies. Annual serological surveillance of infectious diseases would take advantage of the targeted sampling if we can assume that the FOI of target diseases remains similar over time.

To summarize, the optimal target population with the narrowest 95% CI differs depending on the expected FOI. Therefore, sampling should be targeted at the younger age groups to minimize the 95% CI in estimating large FOI. However, sampling should be targeted at the old age groups in estimating small FOI. Our future study will be to justify our strategy using an actual dataset of serological surveillance.

## Data availability statement

The original contributions presented in the study are included in the article/Supplementary material, further inquiries can be directed to the corresponding author.

## Author contributions

KK conducted simulations and the analysis of data. KK and KI wrote the manuscript. All authors contributed to the article and approved the submitted version.

## Funding

This work was supported by the Japan Agency for Medical Research and Development (grant number JP21wm0125008) and Japan Society for the Promotion of Science (grant number 21H03490).

## Conflict of interest

The authors declare that the research was conducted in the absence of any commercial or financial relationships that could be construed as a potential conflict of interest.

## Publisher's note

All claims expressed in this article are solely those of the authors and do not necessarily represent those of their affiliated organizations, or those of the publisher, the editors and the reviewers. Any product that may be evaluated in this article, or claim that may be made by its manufacturer, is not guaranteed or endorsed by the publisher.
