# Supplementary material for: Targeted sampling reduces the uncertainty in force of infection estimates from serological surveillance
Source: Front Vet Sci. 2022 Jul 28;9:754255. doi: 10.3389/fvets.2022.754255 (PMC9366556; doi:10.3389/fvets.2022.754255)
Supplement: Supplementary file 4 [file Presentation_1.pdf]

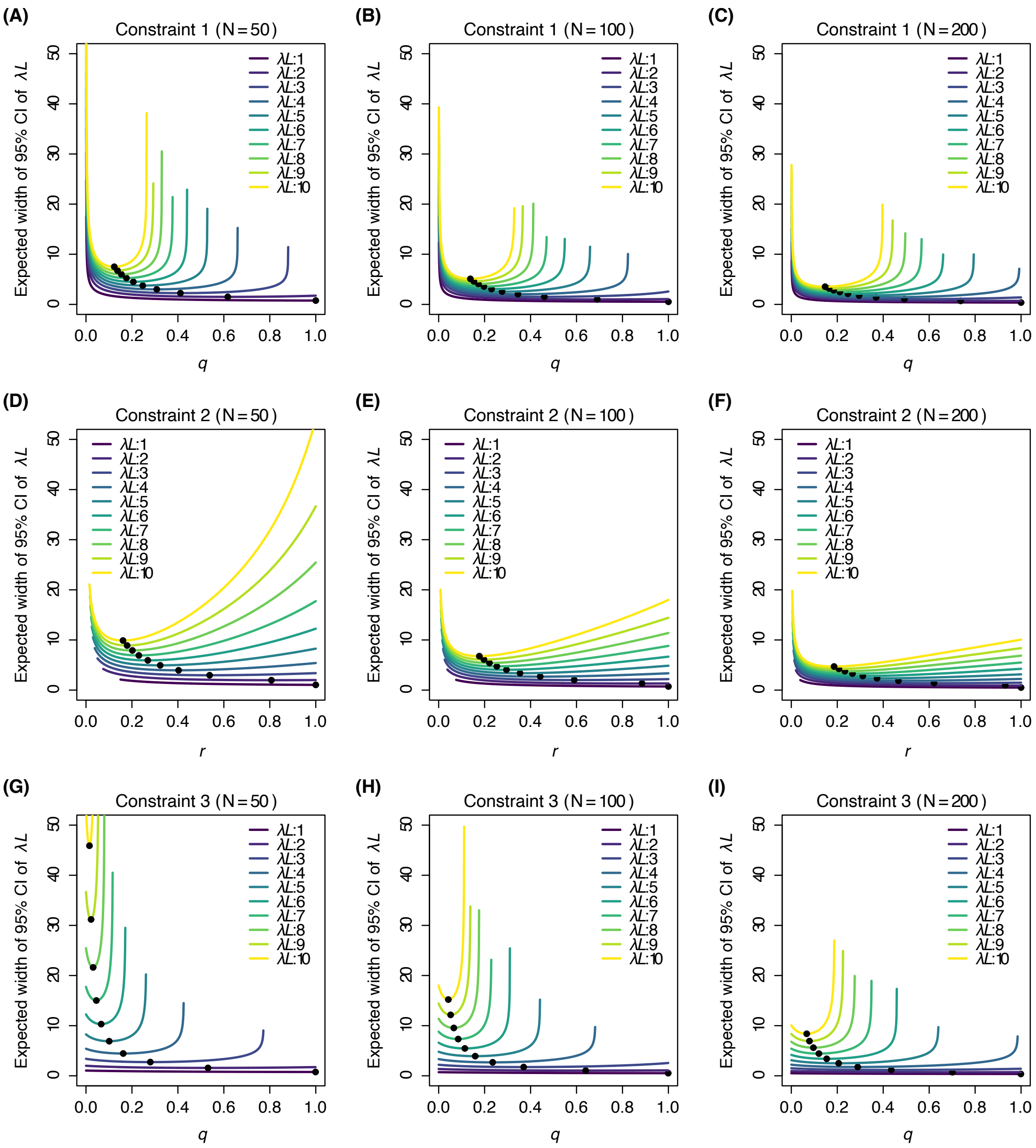

Supplementary Figure 1. Relation between optimum parameter and expected width of 95% CI with different λL and different sample number (N) under Constraint 1 and Constraint 2, and Constraint 3. The lines are color coded by the value of λL shown in the legend. The closed circle denotes the minimum expected width of 95% CI for given λL. The expected width of 95% CI was calculated under setting where was Constraint 1 (Panel (A), (B), and (C)), Constraint 2 (Panel (D), (E), and (F)), and Constraint 3 (Panel (G), (H), and (I)). The expected width of 95% CI was calculated under assumption that N= 50 (Panel (A), (D), and (G)), 100 (Panel (B), (E), and (H)), and 200 (Panel C, F, I).

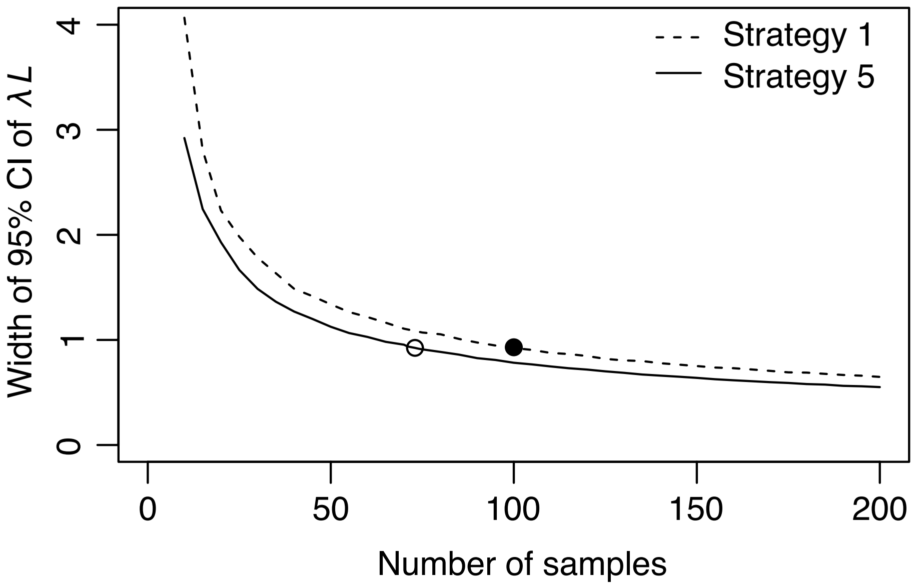

Supplementary Figure 2. Effect of the number of samples in surveillance on the estimation of the width of 95% CI of λL in Strategy 1 and Strategy 5 shown in Table 1. The dashed line represents Strategy 1, and the solid line represents Strategy 5. The number of samples is set to be from 10 to 200. Serological surveillances are simulated 1,000 times under setting where λL= 1.5. The horizontal line represents the width of 95% CI of λL is 0.924. The filled circle denotes the estimated width of 95% CI when the number of samples is 100 under Strategy 1. The circle denotes the estimated width of 95% CI when the number of samples is 73 under Strategy 5.
